# Resveratrol Inhibited ADAM10 Mediated CXCL16-Cleavage and T-Cells Recruitment to Pancreatic β-Cells in Type 1 Diabetes Mellitus in Mice

**DOI:** 10.3390/pharmaceutics14030594

**Published:** 2022-03-09

**Authors:** Mohamed S. Abdel-Bakky, Abdulmajeed Alqasoumi, Waleed M. Altowayan, Elham Amin, Mostafa A. Darwish

**Affiliations:** 1 Department of Pharmacology and Toxicology, College of Pharmacy, Qassim University, Buraydah 52471, Saudi Arabia; m.abdelbakky@qu.edu.sa; 2 Department of Pharmacology and Toxicology, Faculty of Pharmacy, Al-Azhar University, Cairo 11884, Egypt; 3 Department of Pharmacy Practice, College of Pharmacy, Qassim University, Buraydah 52471, Saudi Arabia; a.alqasomi@qu.edu.sa (A.A.); w.altowayan@qu.edu.sa (W.M.A.); 4 Department of Pharmacognosy, Faculty of Pharmacy, Beni-Suef University, Beni Suef 62514, Egypt; el.saleh@qu.edu.sa; 5 Department of Medicinal Chemistry and Pharmacognosy, College of Pharmacy, Qassim University, Buraydah 52471, Saudi Arabia; 6 Department of Pharmacology and Toxicology, Faculty of Pharmacy, Nahda University, Beni Suef 11787, Egypt

**Keywords:** CXCL16, ADAM10, NF-κβ, apoptosis, pancreatic islets, resveratrol, T1D

## Abstract

**Background:** CXCL16 attracts T-cells to the site of inflammation after cleaving by A Disintegrin and Metalloproteinase (ADAM10). **Aim:** The current study explored the role of ADAM10/CXCL16/T-cell/NF-κB in the initiation of type 1 diabetes (T1D) with special reference to the potential protecting role of resveratrol (RES). **Methods:** Four sets of Balb/c mice were created: a diabetes mellitus (DM) group (streptozotocin (STZ) 55 mg/kg, i.p.], a control group administered buffer, a RES group [RES, 50 mg/kg, i.p.), and a DM + RES group (RES (50 mg/kg, i.p.) and STZ (55 mg/kg, i.p.) administered daily for 12 days commencing from the fourth day of STZ injection). Histopathological changes, fasting blood insulin (FBI), glucose (FBG), serum and pancreatic ADAM10, CXCL16, NF-κB, T-cells pancreatic expression, inflammatory, and apoptotic markers were analyzed. **Results:** FBG, inflammatory and apoptotic markers, serum TNF-α, cellular CXCL16 and ADAM10 protein expression, pancreatic T-cell migration and NF-κB were significantly increased in diabetic mice compared to normal mice. RES significantly improved the biochemical and inflammatory parameters distorted in STZ-treated mice. **Conclusions:** ADAM10 promotes the cleaved form of CXCL16 driving T-cells into the islets of the pancreatic in T1D. RES successfully prevented the deleterious effect caused by STZ. ADAM10 and CXCL16 may serve as novel therapeutic targets for T1D.

## 1. Introduction

There are over 37 members of the group of A Disintegrin and Metallopeptidases (ADAMs); among them, ADAM10 and ADAM17 are functionally and structurally similar [1,2]. ADAM10 has an important role in the signaling of Notch and shedding amyloid precursor protein (APP) accompanying Alzheimer’s disease pathophysiology. ADAM10 has a central role in cell migration, cell adhesion, and the regulation of immunity responses, in addition to controlling apoptotic process [3,4]. C-X-C motif chemokine ligand 16 (CXCL16) is an exceptional chemokine existing in two different forms: the soluble CXCL16 binds to immune cells that express its receptor, CXCR6, to drive them into the site of inflammation [5], and the cell membrane form that acts as a receptor then internalizes oxidized low-density lipoprotein (ox-LDL) [6]. ADAM10 and/or ADAM17 can cleave the membranous CXCL16 to its soluble form [7]. 

Our earlier work has revealed that both proteins (CXCL16 and ADAM10) are expressed in the renal glomerular podocytes, principle cells of the distal tubules, collecting duct, and early part of Henle’s loop [8]. In addition, recent work has reported that CXCL16 may have an essential role in the promotion of ovarian cancer [9]. Also, serum levels of CXCL16 were increased in the case of gout, in addition to chronic kidney diseases (CKD), and is accompanied by deterioration of renal function [10,11]. Notably, CXCL16 has an important role in NK and T-cell recruitment [12]. Moreover, it is enhanced by TNF-alpha and IFN-gamma inflammatory cytokines [13].

Tawfik et al., (2021) has shown in a recent study that the CXCL16 serum level was elevated significantly in patients with Type 2 DM, relative to that of normal individuals [14]. Similarly, CXCL16 serum levels in T2DM patients, with or without coronary artery disease, were increased if compared with healthy individuals [15]. Likewise, serum CXCL16 level was found to increase in gestational diabetes mellitus [16]. Interestingly, TNF-α induced apoptosis is enhanced by serum CXCL16 in the DLBCL cell-type; this may comprise serum CXCL16, ADAM10, and the TNF-α mediated NF-κB pathway. However, few records concerning the role of ADAM10 and CXCL16 in the progression of T1D were found. Indeed, the role of ADAM10 in diabetes is still questionable. Abdel-Bakky et al., 2022 found that simvastatin treatment reduced CXCL16 expression in the islets of the pancreas in STZ-treated mice [17]. An earlier report demonstrated that treating 5XFAD mice with STZ did not change ADAM10 levels [18]. Furthermore, an additional report demonstrated significant elevation in ADAM10 protein expression in diabetic minipigs after vascular injury [19]. This work aimed to investigate the role of ADAM10 and CXCL16 in STZ-induced T1D in mice.

Resveratrol (RES) is a phytoalexin polyphenolic compound which exists in berries, grapes, and peanuts. It has anti-inflammatory, anti-oxidant, cardioprotective, antidiabetic, anticancer, and neuroprotective activities [20,21,22]. It may protect against the consequences of diabetes, including nephropathy or cataracts [23,24]. In addition, RES reduces pancreatic inflammatory factors and glucose levels in the serum of cardiovascular-complicated diabetic models of rats [25]. Also, RES showed antidiabetic effects experimentally and clinically through several mechanistic pathways and targets [26]. The aim of the current study is to explore the possible antidiabetic mechanism influencing RES against T1D induced by STZ. Moreover, the interdependence between ADAM10 and CXCL16 in STZ-induced T1D will also be explored.

## 2. Materials and Methods

### 2.1. Work Design

The experimental design and work involved were in agreement with the Institutional Animal Care and Use Committee of the University of Nahda in Egypt (NUB-002-021). BALB/c male albino mice (weighing 23–29 g; supplied by the Nahda animal facility) were given standard lab chow (El-Nasr Company, Cairo, Egypt) and water ad libitum. Mice were accommodated at 22 °C ± 2 °C with 50% ± 10 humidification percentage with a 12 h day-night cycle. Animals were randomly classified into 4 groups (*n* = 10). Normal mice received i.p. sodium citrate buffer. RES animals were administered RES alone (50 mg/kg, i.p.) for 12 days commencing from day 4 until day 15. Diabetic mice (DM) received STZ (55 mg/kg, i.p.), dissolved in a sodium citrate buffer (pH 4.5, 50 mmol/L) once daily for 5 consecutive days [27]. DM + RES group animals received i.p., 55 mg/kg STZ, once daily for 5 consecutive days and RES (50 mg/kg, i.p. dissolved in 25% dimethylsulfoxide, DMSO) for 12 days beginning on day 4 after injecting STZ until the day 15 of the experiment. In the DM + RES group, RES was administered after STZ treatment by 30 min on days 4 and 5 of the experiment. Doses used of RES and STZ injection were guided by our previous work [28,29].

### 2.2. Tissue Samples and Serum Collection

Diethyl ether was used as anesthesia, and mice were euthanized by cervical dislocation on day 15 of the experiment. Collected pancreatic tissues were fixed in Davidson buffer to perform immunofluorescence analysis. Blood samples were collected by small capillary tubes from the retro-orbital plexus before sacrifice. The preparation of the serum was performed by centrifugation of the collected blood at 4000 rpm for 15 min, and the parameters were analyzed using the ELISA technique and blood analyzer.

### 2.3. Pancreatic Islet Isolation

The pancreatic islets were isolated in a separate experiment as previously described [30]. In brief, isolated pancreas was cut into tiny slices and collected in cold collagenase (2 mL, 1.0 mg/mL) in Hank’s balanced salt solution (HBSS; Invitrogen, Waltham, CA, USA) for digestion. Samples were digested for 15 min at 37 °C in an incubator. Then, ice-cold HBSS was mixed into the digested samples, and the islets were separated by Ficoll gradient (Ficoll–Paque Plus; GE Healthcare, Chicago, IL, USA). After a gentle vortex of the suspended mixture using high speed for 10 s, the islets were separated and picked up, aided by an inverted Leica DMIL LED microscope. The separated pancreatic islets were then subjected directly to Western blot analysis.

### 2.4. Estimation of Blood Glucose, Insulin, WBC Count, and Body Weight Change

Blood glucose, white blood cell (WBC) count, and serum insulin were measured in blood samples collected from all groups at the end of the experiment. Direct measurement of blood glucose levels was determined in all groups using a glucometer (Uright, New Taipei City, Taiwan). An ELISA kit (Biovision Inc., Milpitas, CA, USA) was used to determine serum insulin. Furthermore, WBCs were counted using an ABX Micros 60 Analyzer auto blood analyzer (ABX Micros 60, Montpellier, France). The following equation was used to determine changes in body weight: Δ body weight = (weight of animals on day 15 − weight of animals on day 0).

### 2.5. Serum NO Estimation

Estimation of serum NO was accomplished according to the previous method [31]. In brief, 0.5 mL of cold absolute ethanol was mixed with 250 µL serum samples, and the mixture was lifted for 2 days at +4 °C in order to achieve full precipitation of the protein. Samples were then centrifuged at 15,000 rpm for 60 min, 250 µL of the obtained supernatant was mixed with 250 µL of VCl3 and125 µL of sulfanilamide, and 125 µL of *N*-(1-Naphtyl) ethylenediamine dihydrochloride (NEDD) was rapidly added. Samples were incubated at 37 °C for 30 min and the resulting pink chromophore was then analyzed using a double-beam spectrophotometer (UV-150-02, Shimadzu, Toyko, Japan) at 540 nm.

### 2.6. Immunofluorescence Analysis

Paraffin-fixed tissue samples were deparaffinized by dipping the slides in xylene and then in series of ethyl alcohol concentrations for rehydration. After washing the sections in 0.05% Tween 20 in 10 mM phosphate-buffered saline (pH 7.4), antigen retrieval was performed by dipping the slides in sodium citrate buffer (0.01 M, pH 6.0) and boiling them for 15 min in a microwave oven (500 W). To fix or permeabilize tissues, sections were covered for 20 min by drops of 100% methyl alcohol. The blocking step was performed by incubation of the sections with a blocking buffer (BPS containing 1% bovine serum albumin in 10% horse serum) for 60 min. Slides were then washed and incubated with the primary Abs (polyclonal goat anti-mouse CXCL16 or mouse monoclonal ADAM10) overnight in a refrigerator. After washing, the sections were incubated with secondary Abs (rabbit anti-goat Alexa 488 or goat anti-mouse Cy3) for 30 min. Nuclei were counterstained by the incubation of the slides in 4′,6′-diamidino-2-phenylindole (DAPI) for 1 min. Tissue sections were then washed for 30 min and mounted using fluoromount G and analyzed using fluorescence microscopy (Leica DM5000 B, Wetzlar, Germany) [32]. Quantification of the florescence intensities was analyzed by measuring 4 to 6 fields to obtain the mean intensity for each tissue section by ImageJ/NIH software 1.51 (MD, USA).

### 2.7. Immunohistochemistry of Pancreatic Tissue Sections

Tissue samples fixed in paraffin were deparaffinized by dipping the slides in xylene and in a series of ethyl alcohol concentrations for rehydration. After washing sections in buffer (10 mM phosphate-buffered saline containing 0.05% tween 20, pH 7.4), slides were boiled for 20 min in an oven (500 W) in 0.01 M sodium citrate buffer (pH 6.0) for the antigen retrieval step. Blocking endogenous peroxidase was performed by incubating the sections with 3% H_2_O_2_/methanol. The sections were blocked by BPS (containing 1% bovine serum albumin in 10% horse serum) for 60 min and kept with avidin/biotin-blocking kits for 15 min. After incubating the slides with the primary antibody (mouse monoclonal CD3, 1:200 diluted in blocking buffer overnight at 4 °C, Universal Quick kits were used, followed by an AEC Substrate kit, to develop the red color. Nuclei were counterstained with hematoxylin and finally mounted with mounting solution. The pictures were captured by light microscope (Leica DM5000 B, Wetzlar, Germany)

### 2.8. Western Blot Analyses

Western blot analysis was performed in accordance to the method previously described [33]. Ready Prep™ buffer for the extraction of the protein (Bio-Rad Inc., catalog #163-2086, Hercules, CA, USA) was used to determine protein content by Bradford protein assay kit in the pancreatic isolated islet tissue lysates of all the groups. Subsequently, equal concentrations of protein from all samples were mixed with loading buffer (Laemmli) and separated by 10% sodium dodecyl sulfate (SDS)-polyacrylamide gels. Separated protein contents were blotted to nitrocellulose membrane (Millipore, Burlington, MA, USA). The membrane was then blocked using 5% skim milk for 1 hr and incubated with the primary Abs against β-actin, NF-κB, and ADAM10, and then incubated with the secondary Ab (HRP-conjugated goat IgG). Visualization of the proteins was performed using the enhanced chemiluminescent kit. For equal loading, the membrane was incubated with β-actin Ab. Densitometry was performed to semi-quantify the protein bands of β-actin and presented as a bar graph.

### 2.9. Kits, Chemicals, and Antibodies

A mouse insulin ELISA kit was procured from Biovision (Milpitas, CA, USA). Mouse ADAM10, CD3, insulin, cleaved caspases-3, and NF-κB were purchased from Santa Cruz Biotechnology (Dallas, TX, USA). Goat anti-mouse Cy3 and rabbit anti-goat Alexafluor 488 secondary Abs were obtained from Invitrogen (Waltham, CA, USA). The mouse CXCL16 ELISA kit and TUNEL assay kit—HRP-DAB were obtained from Abcam (Cambridge, UK). Goat anti-rabbit polyclonal CXCL16 Ab was obtained from Peprotech (London, UK). STZ, monoclonal mouse against β-actin Ab, RES, and collagenase were purchased from Sigma-Aldrich (St. Louis, MO, USA). RES was purchased from Colchester (Colchester, UK). The Bradford kit for protein assay was purchased from Bio Basic Inc. (Burlington, ON, Canada). Goat IgG secondary antibody-conjugated horseradish peroxidase (HRP) was obtained from Novus Biologicals (Abingdon, UK). ECL substrate (Clarity™) for Western was purchased from Bio-Rad (Hercules, CA, USA).

### 2.10. Statistical Analysis

Data were presented as mean ± SEM and statistically evaluated using one-way ANOVA for multiple group comparisons and Tukey–Kramer as a post-ANOVA test using GraphPad Prism, version 6 (San Diego, CA, USA). The difference between groups was deemed statistically significant at *p* < 0.05.

## 3. Results

### 3.1. Effect of RES on Fasting Blood Glucose, Serum Insulin and Body Weight

**Figure 1:**
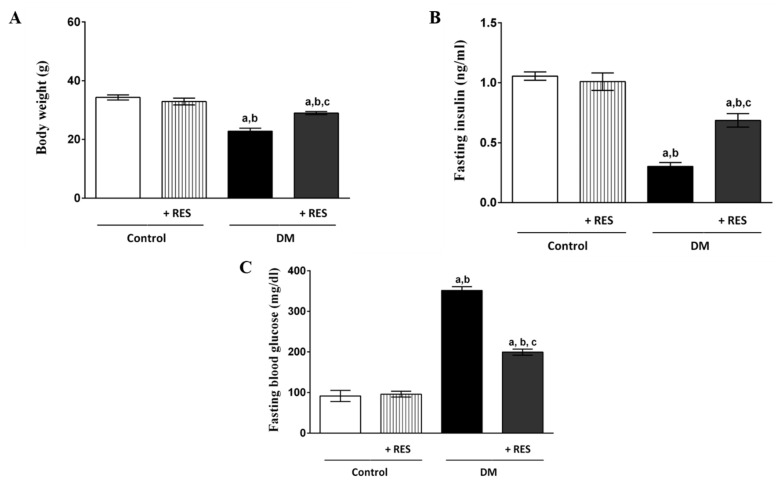
STZ-injected mice showed significant decreases in body weight (**A**) and fasting serum insulin (**B**) compared to control. STZ-injected mice revealed significant elevation in fasting blood glucose level (**C**) when compared to control mice. RES-injected animals showed no difference in fasting blood glucose, serum insulin, or body weight compared to control group. Remarkably, significant improvement was seen in levels of fasting blood glucose, body weight, and fasting serum insulin in RES-treated animals in the presence of STZ. Data represent mean ± SEM: ^a^ difference is significant compared to normal group; ^b^ difference is significant compared to RES group; ^c^ difference is significant compared to diabetic group at *p* < 0.05.

STZ-injected mice had significantly lower body weights and serum insulin levels (Figure 1A,B, respectively) compared to the control group. However, STZ-injected mice demonstrated significant elevation in the level of fasting blood glucose (Figure 1C) compared to normal mice. On the other hand, RES-injected animals exhibited non-significant differences in serum insulin, body weight, and fasting blood glucose when compared to the control group. Remarkably, significant improvement in the above-mentioned parameters was found in treatment mice with RES in STZ-treated mice (Figure 1A–C).

### 3.2. Effect of RES with or without STZ on WBCs, NO or TNF-α

**Figure 2:**
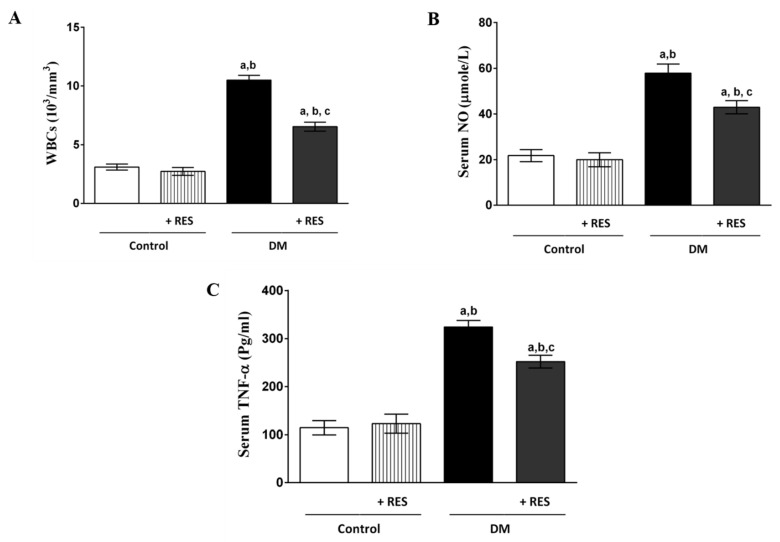
Levels of WBCs (**A**), NO (**B**), and TNF-α (**C**) are significantly increased (approximately 3-fold) in mice injected with STZ compared with normal or RES treatment mice. STZ-treated group injected with RES display significant decreases in WBC count, serum level of NO, and serum level of TNF-α when compared to STZ-treated mice. No significant change in WBC count, serum level of NO, and serum level of TNF-α was seen in RES group compared to control group. Data represent mean ± SEM: ^a^ difference is significant compared to control group; ^b^ difference is significant compared to RES group; ^c^ difference is significant compared to diabetic group at *p* < 0.05.

Increased (by approximately 3-fold) levels of WBCs, NO, and TNF-α were observed in mice injected with STZ compared with normal controls. Mice injected with RES displayed no significant changes in the level of WBCs, TNF-α, or NO when compared to the control group. RES treatment significantly reduced WBC count, serum levels of NO, and TNF-α when compared to diabetic mice (Figure 2A–C).

### 3.3. Effect of RES on STZ-Induced CXCL16 Pancreatic Expression

**Figure 3:**
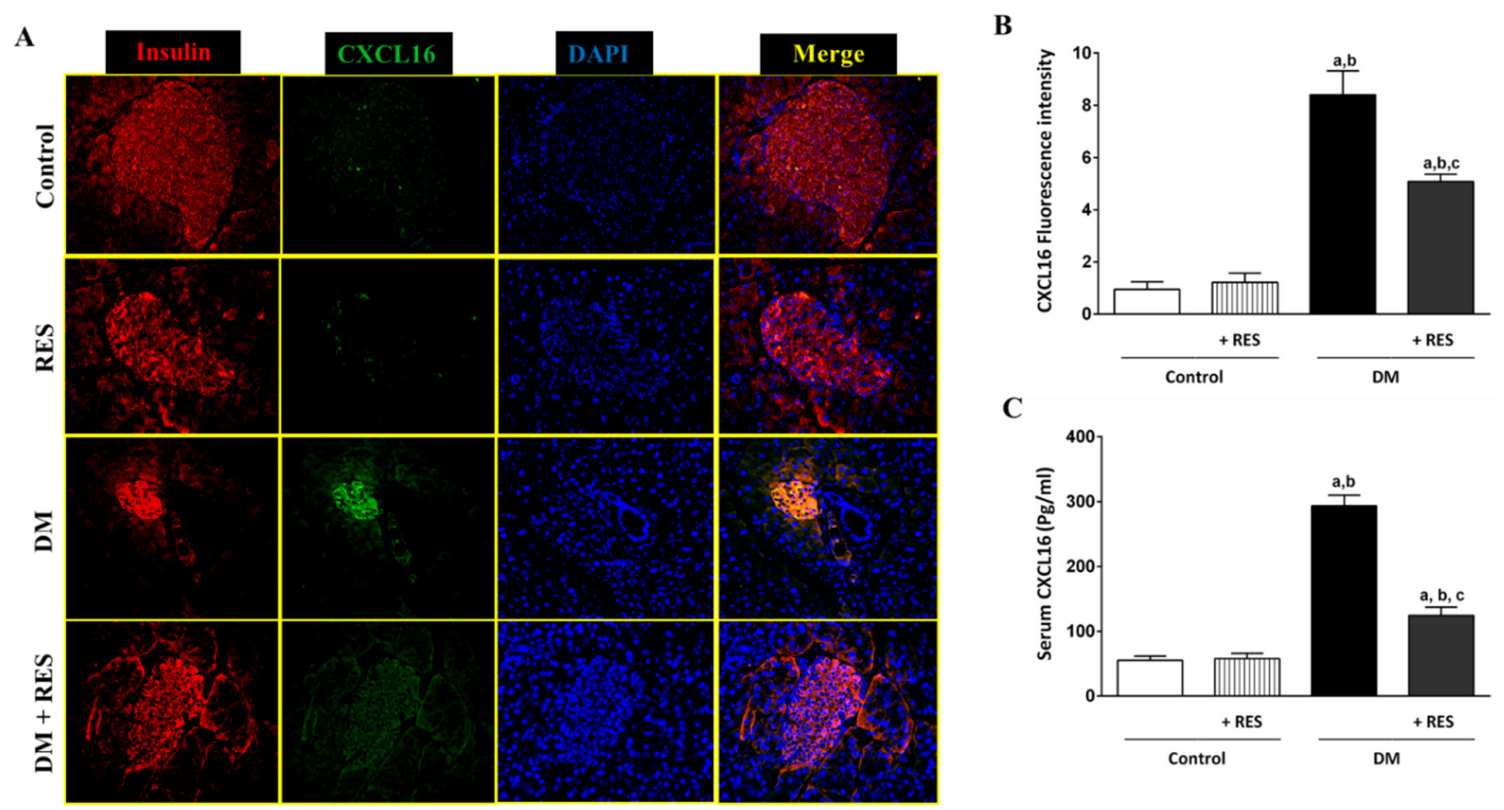
(**A**) Double immunofluorescence staining for insulin (red), a pancreatic β cell marker, and CXCL16 (green) was performed; basal expression of pancreatic β cell CXCL16 in normal control and RES-treated mouse tissues. Increased CXCL16 expression in pancreatic β cells was confirmed by the co-localization of CXCL16 with insulin. Marked reduction of CXCL16 expression in mouse pancreatic β cells of RES-treated mice with STZ was seen; (**B**) Fluorescence intensity of CXCL16 protein expression for all groups was quantified and blotted; (**C**) Serum levels of CXCL16 using ELISA in all treated groups show similar findings of immunofluorescence. Data represent mean ± SEM: ^a^ difference is significant compared to control group; ^b^ difference is significant compared to RES group; ^c^ difference is significant compared to diabetic group at *p* < 0.05.

Using double immunofluorescence staining for insulin, pancreatic β cell marker, and CXCL16, we observed basal expression of CXCL16 in normal control mouse tissues. Treating the mice with RES showed a non-significant difference in the expression of CXCL16 protein in comparison with control mice. Increased pancreatic β cell expression of CXCL16 was seen confirmed by CXCL16 co-localization with insulin (β cell marker). Significant reduction in CXCL16 expression was found in mice pancreatic β cells of diabetic mice treated with RES (Figure 3A). Fluorescence intensity of CXCL16 protein expression for all groups was quantified and blotted, as shown in Figure 3B. Similar findings were found with serum CXCL16 as measured by ELISA (Figure 3C).

### 3.4. Effect of RES on STZ-Induced Pancreatic ADAM10 Expression

**Figure 4:**
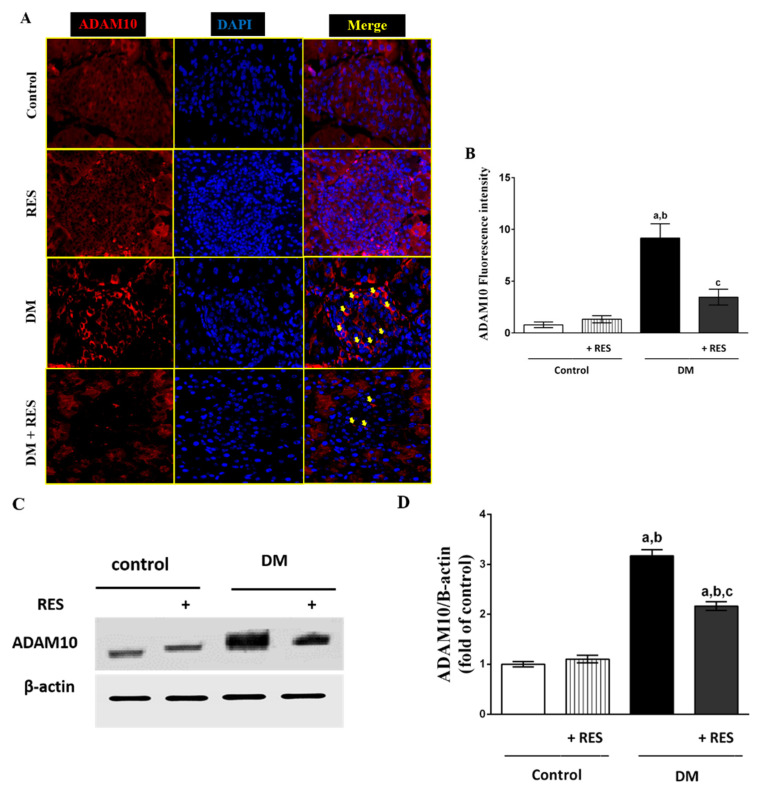
(**A**) Increased expression of ADAM10 protein in the islets of pancreases of STZ-injected mice compared to control group. Decreased expression of ADAM10 in pancreatic β cells after treatment with RES increased by STZ-injection; (**B**) Fluorescence intensity analysis of ADAM10 protein expression from immunofluorescence was quantified and blotted in all experimental groups; (**C**) Expression of ADAM10 protein was analyzed using Western blot in all treated animals. No difference was seen between control and RES-treated groups. STZ-injected animals showed marked increases in ADAM10 protein expression in comparison with the normal group. Animals injected with RES and STZ showed marked reduction in the expression of ADAM10 in islets of pancreas compared to STZ-injected group; (**D**) Densiometry for Western blot bands for ADAM10 in all groups was performed using NIH ImageJ software (MD, USA). Data represent mean ± SEM: ^a^ difference is significant compared to control group; ^b^ difference is significant compared to RES group; ^c^ difference is significant compared to diabetic group at *p* < 0.05.

Consistent with CXCL16 expression in β cell islets, ADAM10 upregulation in the pancreatic islets was found in animals treated with STZ in comparison with control animals. However, RES decreased pancreatic β cell expression of ADAM10 increased by STZ-injection (Figure 4A). Fluorescence intensity analysis of ADAM10 protein expression from immunofluorescence was quantified and blotted, as shown in Figure 4B. For more confirmation, expression of ADAM10 protein was analyzed by Western blot in all treated animals. As shown in Figure 4C, no difference was found between control and RES-treated mouse tissues. On the other hand, STZ-treated mice demonstrated a marked elevation in ADAM10 protein expression as compared to control mice. Reduced ADAM10 protein expression was detected in the [RES + STZ] group in comparison with diabetic mice. Densiometry for Western blotting bands for ADAM10 was performed using NIH ImageJ software (Figure 4D)

### 3.5. Expression of NF-κB Protein in the Islets of Pancreas in RES-Injected Mice with or without STZ

**Figure 5:**
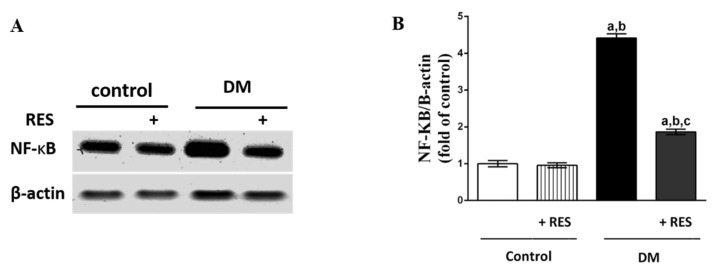
(**A**,**B**) Expression of NF-κΒ in β cells was significantly increased in STZ-injected animals in comparison with normal mice. RES treatment significantly reduced the expression level of NF-κΒ of STZ-treated group in comparison with the STZ-injected group. No change was found in NF-κΒ expression in RES-injected mice in comparison with the normal group. Data represent mean ± SEM: ^a^ difference is significant compared to control group; ^b^ difference is significant compared to RES group; ^c^ difference is significant compared to diabetic group at *p* < 0.05.

Increased NF-κΒ expression in the islets of the pancreas was found in STZ-injected animals in comparison with normal mice, as shown by Western blot analyses. Treatment with RES in the STZ group revealed a marked reduction in the expression of NF-κΒ protein in comparison with the STZ-injected mice. No marked changes in NF-κΒ protein expression were found in the RES-injected group in comparison with normal animals (Figure 5A,B).

### 3.6. Effect of RES on STZ-Induced Apoptosis

**Figure 6:**
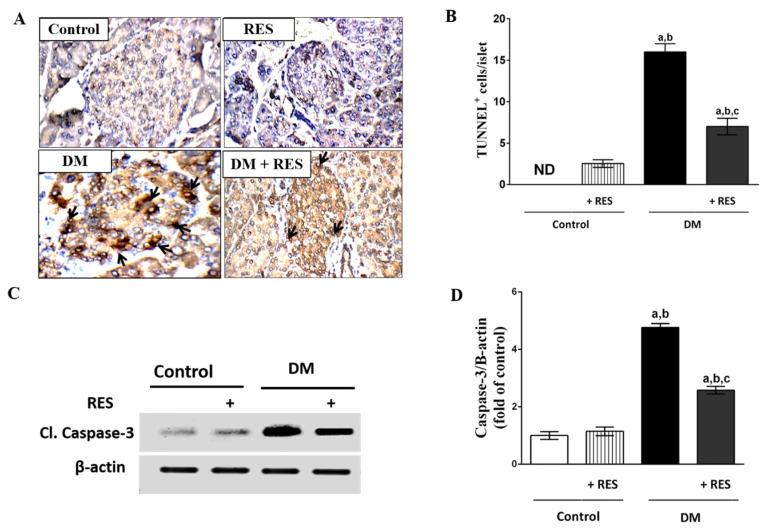
(**A**,**B**) Pancreas of normal mice demonstrates negative TUNEL reactivity in the nucleus in islet cells. In RES group, strong reactivity in the nucleus in few cells/ islets for TUNEL was seen. On the other hand, islets of pancreas of STZ-injected mice revealed strong reactivity in the nucleus for TUNEL that was higher in number of cells in comparison with the normal islets. RES and STZ-injected group displayed mild reactivity in the nucleus for TUNEL in limited cell numbers when compared with mice treated with STZ; (**C**,**D**) Expression of pro-apoptotic protein, cleaved caspase-3, increased significantly in STZ treatment in pancreatic islets of mice using Western blot analysis compared to normal mice. Animals injected with RES with STZ displayed mild reactivity in the nucleus for TUNEL in few cells in comparison with the STZ-treated mice. No marked change in the expression of caspase-3 protein in the RES treatment group was noted compared to the normal group. Data represent mean ± SEM: ^a^ difference is significant compared to control group; ^b^ difference is significant compared to RES group; ^c^ difference is significant compared to diabetic group at *p* < 0.05.

Pancreatic sections of the control group showed negative reactivity in the nucleus for TUNEL in the cells of the pancreatic islets while in the RES group; strong reactivity in the nucleus in a few cells/ islets for TUNEL was seen. On the other hand, the islets of the pancreas of STZ-injected mice displayed a strong reactivity in the nucleus for TUNEL that was higher in number of cells in comparison with the normal islets. Animals injected with RES with STZ displayed mild reactivity in the nucleus for TUNEL in few cells in comparison with the STZ-treated mice (Figure 6A,B).

For more confirmation of the apoptotic pathway, expression of caspase-3 was also tested in isolated islets. The expression of pro-apoptotic protein, cleaved caspase-3, increased significantly in STZ treatment in the pancreatic islets of mice using Western blot analysis compared with normal mice, while mice injected with RES together with STZ displayed a reduction in the level of expression of cleaved caspase-3 in the pancreatic islets as compared to diabetic mice. RES-treated mice showed a similar expression of caspase-3 to that of the control group (Figure 6C,D).

### 3.7. Effect of RES with or without STZ on Splenic and Pancreatic T-Cell Protein Expression

**Figure 7:**
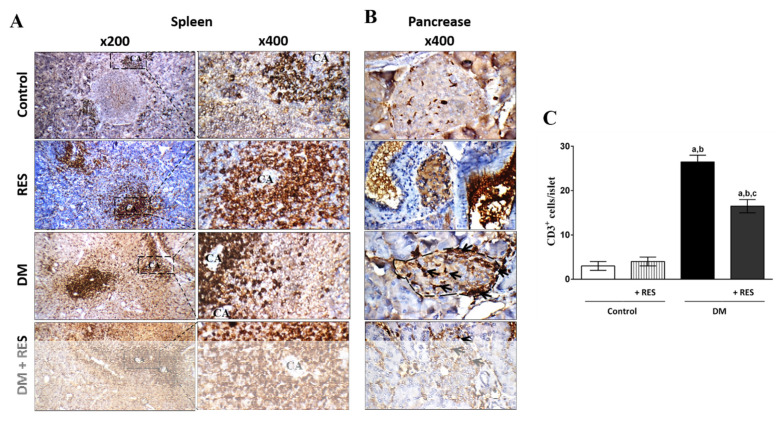
(**A**) Spleen of STZ-treated mice shows marked increase in CD3 positive cells in peri-arteriolar area in certain follicles and high reactivity in red bulb area in comparison with the average positive cells for CD3 in peri-arteriolar area (T-cell area) with mild reactivity in red bulb in normal spleen of the control or RES groups. However, STZ-injected group in the presence of RES displayed spleen with average positive cells for CD3 in peri-arteriolar area and mild reactivity in red bulb area (400× and 200×) compared to STZ-treated group; (**B**,**C**) Normal and RES-injected animals show normal number of positive cells for CD3/ islet. Pancreatic islets from STZ-injected mice display marked increase in CD3 positive cells/islets in comparison with normal animals. Diabetic group injected with RES reveals marked decrease in positive cells for CD3/islets in comparison with STZ-injected animals, as shown by immunohistochemistry and the average count of T-cells/islets for all treated groups (400×). Data represent mean ± SEM; ^a^ difference is significant compared to control group; ^b^ difference is significant compared to RES group; ^c^ difference is significant compared to diabetic group at *p* < 0.05.

The spleens of diabetic mice showed marked increases in CD3 positive cells in the peri-arteriolar area in some follicles and high reactivity in the red bulb area in comparison with the average positive cells for CD3 in the peri-arteriolar area (T-cell area) with mild reactivity in the red bulb in the normal spleen of the control or RES-treated groups. However, the STZ-injected group in the presence of RES displayed spleens with average CD3 positive cells in the peri-arteriolar area and mild reactivity in the red bulb area (Figure 7A).

In addition, the pancreases of the control animals revealed normal positive numbers of CD3 cells/islet. Moreover, no significant difference in CD3 positive cells numbers in the islets of pancreas of RES treated animals in comparison with an increase in normal islets. In contrast, islets of pancreas of STZ-injected mice displayed marked in CD3 positive cells/islets in comparison with normal animals. Diabetic mice injected with RES showed marked decrease in the positive CD3 cells/islets in comparison with STZ-treated mice as shown by CD3 positive T-cell expression using immunohistochemistry (Figure 7A) and the average number of T-cells/islets for all treated groups (Figure 7B).

### 3.8. RES and/or STZ Effect on Histopathological Features

**Figure 8:**
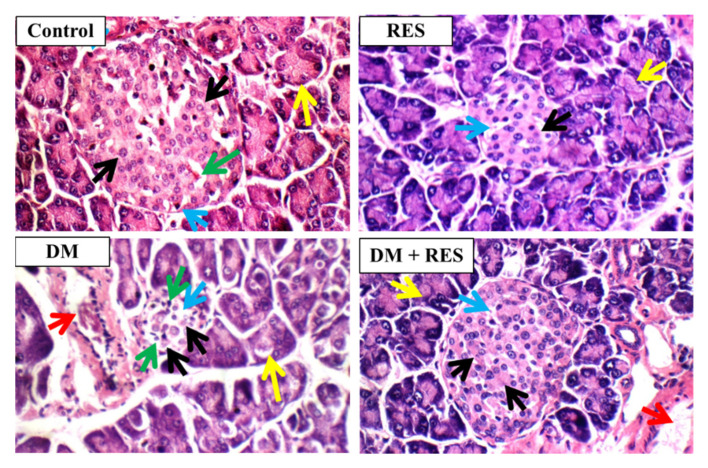
Islets of pancreas from control and RES-injected mice showed average islets, fewer α cells, marginal pink cytoplasm (blue arrows), average dominant thin-wall blood capillaries (green arrows), normal exocrine spaces (yellow arrows), and predominating β-cells with middle-pale-blue cytoplasm (black arrows). Pancreatic tissue of mice treated with STZ shows obvious apoptotic β-cells (black arrows); noticeable dilated principal blood capillaries (blue arrows); inflammatory infiltrate (green arrows); noticeable small-sized hypo-cellular islets, average exocrine zones (yellow arrows); and congested interstitial blood vessels (red arrows). Section from pancreas of mice injected with RES and STZ displays normal-sized islets and a low number of dispersed apoptotic β-cells (black arrows) and normal exocrine spaces (yellow arrows), normal prevailing blood capillaries (blue arrows), and normal blood vessels (red arrows) (H&E × 400).

The islets of the pancreas from the normal group showed normal islets with normal β cells, a low number of α cells with pink cytoplasm in the border (blue arrows), pale blue cytoplasm in the center (black arrows), normal dominant thin-wall capillaries (green arrow), and normal exocrine regions (yellow arrows). RES-injected mice exhibited relatively small-size islets with low distributed apoptotic β cells (black arrows) with mildly dilated intervening capillaries (blue arrows), and normal exocrine zones (yellow arrows). Animals injected with STZ demonstrated noticeable small-size, hypo-cellular islets and clear apoptotic β cells (black arrows), apparent dilated dominant blood capillaries (blue arrows), inflammatory infiltrate (green arrows), and interstitial congested blood vessels (red arrows) with normal exocrine spaces (yellow arrows). However, the RES and STZ-injected group displayed islets with a normal size with a low number of dispersed apoptotic β cells (black arrows), normal interstitial blood vessels (red arrows) and average exocrine parts (yellow arrows) (H&E × 400) (Figure 8).

## 4. Discussion

Diabetes mellitus is a common metabolic disease characterized by an imbalance between the resource and consumption of glucose at the cellular level [34]. The exact mechanism underlying the development of this disease is still unclear. Increased oxidative stress, inflammation, and apoptosis during T1D may lead to pancreatic β-cell loss [35,36]. In addition, T-cell infiltration into pancreatic β-cells mediates inflammatory cascade and consequently β-cell loss [37]. A previous study reported that weight loss is a common finding in diabetes induced by STZ injection that may be due to the degeneration of adipocyte and muscle tissues in order to recompense energy loss [38]. Furthermore, STZ in a high, single dose causes β-cell death due to necrosis and consequently T1D [39]. However, STZ with low multiple doses causes partial β-cell destruction and generates an inflammatory process [40]. The current work was designed to investigate the possible protecting effect of RES in T1D and the possible mechanism(s) underlying this protection, in addition to determining the role of CXCL16 and ADAM10 in the initiation of T1D. Recorded results demonstrate that RES prevents T1D in mice through the attenuation of CXCL16/ADAM10/TNF-α/NF-κB pathway, T-cell infiltration, and caspase-3 mediated apoptosis in the pancreatic islets. To our knowledge, this is the first work exploring the protective effect of RES in term of its effects on CXCL16/ADAM10-mediated pancreatic islets apoptosis, inflammation, and T-cell infiltration in T1D pathogenesis.

In T1D, the increased fasting blood glucose level, reduced serum insulin level, and reduction of animal weight are essential markers of diabetes [41]. Accordingly, the recorded significant weight loss, increased fasting blood glucose level, decreased fasting blood insulin level, obvious small-size hypo-cellular islets, and clear apoptotic β-cell destruction confirmed the successful induction of diabetes with low multiple doses of STZ (55 mg/kg i.p. for 5 consecutive days) in mice. The current findings reveal that RES prevented weight loss, which could be due to the glucose-lowering effect of RES. In addition, RES ameliorated the reduced serum level of insulin caused by STZ injection and prevented β-cell damage, as shown by histopathological results with subsequently increased insulin secretion and glucose utilization.

TNF-α is one of the important serum pro-inflammatory cytokines [42]. Increased serum levels of TNF-α have been accompanied by diabetes development, as it is known to interfere with signal transduction mediated by insulin receptor that leads to the inhibition of the insulin effect [43,44]. Oxidative stress and metabolic disturbance have been reported in T1D [45,46]. In addition, increased oxidative stress in the pancreatic islets is known to be an important pathological mechanism of β-cell damage in an STZ animal model [47,48]. The results of the current study reveal that RES inhibits TNF-α, WBC count, and serum NO-induced by STZ injection. Lin et al., (2019) reported that RES downregulated the expression of monocyte chemoattractant protein-1 (MCP-1) via TNF-α- inhibition in rats with acute pulmonary thromboembolism [49]. In addition, RES-attenuated renal injury resulted from ischemia/reperfusion (I/R) in diabetic rats through TNF-α-stimulated inflammation and oxidative stress [50]. Additionally, RES decreased hepatic I/R injuries associated with increased liver tissue levels of TNF-α and oxidative stress in diabetic rats [51].

Being an exceptional chemokine, CXCL16 exists in two forms: the transmembrane that acts as a receptor for ox-LDL [52] and the soluble form resulting from cleavage of its transmembrane form by ADAM (ADAM10 or ADAM17), and consequently, the released part can attract CXCR6-expressing T-cells to the site of injury [53]. On this basis, we reported the involvement and the role of the chemokine CXCL16 with its processing enzyme, ADAM10, for the first time, as well as its associated signaling mechanism in the development of T1D. For the pancreatic localization of CXCL16, double immunofluorescence of CXCL16 and insulin (β-cell marker) was performed. In addition, ADAM10 protein expression using immunofluorescence and Western blot analysis of isolated pancreatic islets were also accomplished. Our findings display a marked CXCL16 protein upregulation in β-cells of the pancreas and increase of its serum level in T1D induced by STZ. Similarly, increased expression of ADAM10 protein in β-cells of the pancreas was confirmed, adopting immunofluorescence and Western blot analysis. Our previous work confirmed that the activation of CXCL16/ox-LDL pathway in beta cells is a possible cause of TF activation and autophagy of the pancreatic beta cell in T1D [54]. Similarly, CXCL16 promotes inflammatory markers and infiltrated cell factors, and reduces antioxidants, subsequently accelerating diabetic nephropathy development [55]. Also, simvastatin alleviated STZ-induced ADAM10 and CXCL16 in diabetic mice [17].

NF-κB mediates β-cell inflammation resulting in the recruitment of immune cells and insulitis development that mediates T1D development [56]. Abu El-Asrar et al. (2021) suggested that CXCL16/CXCR6 and the processing enzyme ADAM10 play a role in the initiation and progression of proliferative diabetic retinopathy [57]. The author mentioned that the proliferative diabetic retinopathy mediated through increased NF-κB, VEGF and ICAM-1 in the retina. Similarly, regulation of CXCL16 and ADAM10 expression is considered to be an early response in diabetic nephropathy development [52]. Interestingly, a recent report showed that the CXCL16-regulated feedback mechanism is critical for the activation of NF-κB in prostate cancer cells [58]. The role of CXCL16 in the recruitment of T-cells has been described in inflammatory valvular heart disease [59], psoriasis pathogenesis [60], rheumatoid joints [61], colorectal cancer [62], hepatic ischemia-reperfusion injury [63], and interstitial rejection of renal allograft [8]. In the current study, we observed a significant increase in T-cell recruitment to the pancreatic islets as compared with control mice. The recruitment of T-cells in our study is accompanied by ADAM10 upregulation in the pancreatic islets, indicating that ADAM10 may cleave CXCL16 into its soluble form, resulting in an accumulation of T-cells. Notably, the current results show that RES treatment significantly reduces ADAM10 and CXCL16 expressions in the pancreatic β-cells. This inhibition may be explained by the fact that RES may inhibit the membranous level and consequently the ability of ADAM10 to cleave the cellular CXCL16, therefore reducing T-cell recruitment from the blood vessels into the pancreatic β-cells.

Remarkably, an earlier report suggested that apoptosis is induced by CXCL16 in colorectal cancer liver metastasis [64]. Furthermore, ROCK1 activated by CXCL16 causes upregulation of caspase-3 in injured HK-2 cells [6]. Similarly, CXCL16 attracts circulating inflammatory cells to the injured kidney, leading to tubular epithelial-cell apoptosis after cisplatin injection [65]. In support of these results, our findings demonstrate that STZ-induced T1D increased the cellular and soluble form of CXCL16, which mediates apoptosis, as confirmed by cleaved caspases-3 induction, and further confirmed by increased DNA fragmentation shown by TUNNEL assay. Treatment of mice with RES ameliorated the effect of STZ on apoptosis consequences.

## 5. Conclusions

The current study demonstrated for the first time that ADAM10/CXCL16/NF-κB mediated STZ-induced diabetes. Additionally, cleaved CXCL16 mediated T-cell recruitment into β-cells, and therefore enhanced oxidative stress, inflammatory response, and apoptosis. RES successfully mitigated CXCL16 cleavage by ADAM10 and markedly improved the deleterious effects induced by STZ treatment.

## Data Availability

Not applicable.

## Abbreviations

ADAM10	A Disintegrin and Metallopeptidase 10
ANOVA	Analysis of variance
CKD	chronic kidney diseases
CXCL16	CXC chemokine ligand 16
DAPI	4′,6′-diamidino-2-phenylindole
DMSO	Dimethylsulfoxide
ELISA	Enzyme-linked immunosorbent assay
HBSS	Hank’s balanced salt solution
NF-ΚB	Nuclear factor-kappa B
PBS	Phosphate-buffered saline
RES	Resveratrol
STZ	Streptozotocin
TNF-alpha	Tumor necrosis factor-alpha
T1D	Type 1 diabetes
WBC	White blood cell
